# PNA clamping-assisted fluorescence melting curve analysis for detecting *EGFR* and *KRAS* mutations in the circulating tumor DNA of patients with advanced non-small cell lung cancer

**DOI:** 10.1186/s12885-016-2678-2

**Published:** 2016-08-12

**Authors:** Ji-Youn Han, Jae-Jin Choi, Jin Young Kim, You Lim Han, Geon Kook Lee

**Affiliations:** 1 Lung Cancer Branch, Research Institute, National Cancer Center, Goyang, Korea; 2 PANAGENE Inc., Daejeon, Korea; 3 Center for Lung Cancer, Hospital, National Cancer Center, 323 Ilsan-ro, Ilsan-dong-gu, Goyang, Gyeonggi 10408 Korea

**Keywords:** EGFR, KRAS, Plasma, NSCLC, Tissue

## Abstract

**Background:**

Circulating cell-free DNA (cfDNA) is emerging as a surrogate sample type for mutation analyses. To improve the clinical utility of cfDNA, we developed a sensitive peptide nucleic acid (PNA)-based method for analyzing *EGFR* and *KRAS* mutations in the plasma cfDNA of patients with advanced non-small cell lung cancer (NSCLC).

**Methods:**

Baseline tissue and plasma samples were collected from treatment-naïve advanced NSCLC patients participated in a randomized phase II study, which was registered with ClinicalTrials.gov at Feb. 2009 (NCT01003964). *EGFR* and *KRAS* mutations in the plasma cfDNA were analyzed retrospectively using a PNA clamping-assisted fluorescence melting curve analysis. The results were compared with those obtained from tissue analysis performed using the direct sequencing. Exploratory analyses were performed to determine survival predicted by the plasma and tissue mutation status.

**Results:**

Mutation analyses in matched tissue and plasma samples were available for 194 patients for *EGFR* and 135 patients for *KRAS*. The mutation concordance rates were 82.0 % (95 % confidence interval [CI], 76.5–87.4) for *EGFR* and 85.9 % (95 % CI, 80.1–91.8) for *KRAS*. The plasma *EGFR* mutation test sensitivity and specificity were 66.7 % (95 % CI, 60.0–73.3) and 87.4 % (95 % CI, 82.7–92.1), respectively, and the plasma *KRAS* mutation test sensitivity and specificity were 50.0 % (95 % CI, 41.6–58.4) and 89.4 % (95 % CI, 84.2–94.6), respectively. The predictive value of the plasma EGFR and KRAS mutation status with respect to survival was comparable with that of the tissue mutation status.

**Conclusions:**

These data suggest that plasma *EGFR* and *KRAS* mutations can be analyzed using PNA-based real-time PCR methods and used as an alternative to tumor genotyping for NSCLC patients when tumor tissue is not available.

**Electronic supplementary material:**

The online version of this article (doi:10.1186/s12885-016-2678-2) contains supplementary material, which is available to authorized users.

## Background

The paradigm of diagnosis and treatment for advanced non-small cell lung cancer (NSCLC) has changed since epidermal growth factor receptor (EGFR) mutations were identified as the best predictive biomarkers for EGFR-tyrosine kinase inhibitor (TKI) efficacy [1, 2]. Decisions on first-line treatments are based on the target oncogenes identified in tumor tissues; thus, the tumors of patients with NSCLC should be tested for *EGFR* mutations to determine whether an EGFR-TKI is the appropriate first-line therapy [3]. However, obtaining adequate tissue samples for molecular testing can be difficult. Consequently, efforts have been made to evaluate surrogate sample types for molecular testing [4].

Circulation cell-free (cf) DNA in the plasma of cancer patients offers an easily obtainable and repeatedly available source of biological material for mutation analyses [5, 6]. Several methods have been reported for the detection of *EGFR* mutations in cfDNA isolated from plasma, including high performance liquid chromatography, allele-specific polymerase chain reaction (PCR) with Scorpion amplification, peptide nucleic acid (PNA)-mediated PCR clamping, BEAMing, droplet digital PCR (ddPCR), and next-generation sequencing (NGS) [7–9]. The pooled sensitivity and specificity of *EGFR* mutations in cfDNA has been reported at 67.4 and 93.5 %, respectively [10]. Compared with EGFR mutations, KRAS mutations are predictive for a lack of benefit from EGFR-TKI therapy. Although erlotinib has been approved for second or third–line therapy for advanced NSCLC irrespective of *EGFR* mutation status, many studies have demonstrated that patients with *KRAS* mutations show inferior outcomes compared with those with wild-type *KRAS* [11–13]. Thus, *KRAS* genotyping can be considered for patients scheduled to receive EGFR-TKI therapy. Recent data indicate that the cfDNA in plasma could represent a new sample type for the analysis of *KRAS* mutations in tumors and act as a potential biomarker for anti-EGFR therapy efficacy in colorectal cancer [14–17].

PANAMutyper™ R *EGFR* and *KRAS* kits are newly developed sensitive kits that apply a PNA clamping-assisted fluorescence melting curve analysis to perform mutation detection and genotyping. The PNA clamp-assisted melting curve method can discriminate mutant from wild-type alleles through a relatively large melting temperature difference and has a sensitivity of 0.1–0.01 % [18, 19]. Additionally, this method can easily detect the presence of a mutant sequence without an additional data analysis process. The objective of this study was to compare the plasma analysis performed using PANAMutyper™ R *EGFR* and *KRAS* kits with tumor tissue analysis performed using routine *EGFR* and *KRAS* mutation tests. The aim of this analysis was to validate the use of cfDNA as a surrogate sample type for the detection of *EGFR* and *KRAS* mutations in advanced NSCLC.

## Methods

### Patients

Patients with advanced NSCLC who participated in a randomized phase II study that compared gemcitabine and cisplatin (GP) with irinotecan and cisplatin (IP) as first-line therapies were tested in this study. The trial is registered with ClinicalTrials.gov (NCT01003964). The main eligibility criteria included histologic confirmation of advanced NSCLC, no prior chemotherapy, age ≥ 18 years, an Eastern Cooperative Oncology Group (ECOG) performance status (PS) less than 2, and measurable disease according to the Response Evaluation Criteria in Solid Tumors (RECIST). Adequate organ function was required. All of the patients who received at least one cycle of chemotherapy were considered assessable for the progression-free survival (PFS), overall survival (OS), and safety. Archival plasma and tissue samples obtained prior to treatment were used for the *EGFR* and *KRAS* mutation tests. All patients provided written informed consent for the provision of tumor and plasma samples for the biomarker analysis. The protocol was approved by the National Cancer Center Institutional Review Board (study ID number: NCCCTS08371) and conducted in accordance with the Declaration of Helsinki, Good Clinical Practice.

### Clinical assessment

The tumors were assessed by computed tomography of the targeted lesions every two cycles of therapy, every 6 weeks during chemotherapy and every 8 weeks during EGFR-TKI therapy. The objective tumor response was determined using RECIST software, version 1.0 [20]. The PFS was calculated from the start date of each therapy to the date of tumor progression or death. The OS was calculated from the start date of first-line therapy to the date of death or last follow-up.

### *EGFR* and *KRAS* mutation analysis using tumor tissues

Genomic DNA was extracted from 10 % neutral buffered formalin-fixed, paraffin-embedded (FFPE) tumor tissue blocks using the QIAamp DNA Mini Kit (QIAGEN, Hilden, Germany). We analyzed *EGFR* and *KRAS* mutations using the polymerase chain reaction (PCR)-based direct DNA sequencing method [21].

### Plasma DNA extraction and mutation analysis

Circulating cell-free DNA (cfDNA) was extracted from the plasma using the QIAamp Circulating Nucleic Acid Kit (Qiagen, Hilden, Germany). The assays used to detect 47 different *EGFR* variants and 29 *KRAS* variants were obtained using the PANAMutyper™ R EGFR and KRAS kit (Panagene, Daejeon, Korea).

**Fig. 1:**
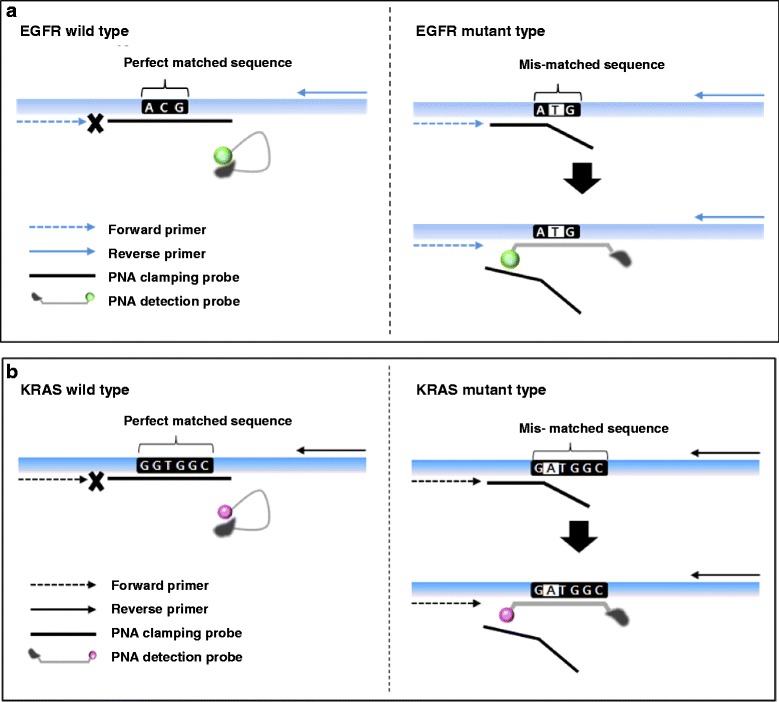
Schematic representation of EGFR and KRAS mutation detection using PANAMutyper^TM^: EGFR (**a**) and KRAS (**b**). Sensitivity of the EGFR L858R and E19del (**c**) and KRAS G12V and G12R (**d**) mutants according to their cellularity by diluting to 100, 10, 1, 0.1, 0.01, 0 % with respect to the wild cell line DNA and mutant cell line DNA. The data presented here are representative obtained from sensitivity test conducted more than 50 times. MT, mutant type

The PNA clamp-assisted melting curve method, the real-time genotyping of somatic mutations, can detect multiple mutations and analyze both curves in a single PCR experiment by using a PNA clamp and PNA detection probe [18, 19, 22]. A detection probe was designed for competitive hybridization with clamping PNA on the same strand (either sense or antisense DNA strand). In our system, two complementary PNA oligomers were designed to hybridize with either a sense or antisense strand (Fig. 1a, b). The PNA detection probes produce an amplification curve and are used to analyze the melting curve. When we take a look closely, all reactions were performed in total volumes of 25 μ l containing 10–25 ng template DNA, primers, PNA probe set, and PCR master mix. The PCR assay was performed under the following conditions: two holding periods of 50 °C for 2 min and 95 °C for 15 min; (i) 15 cycles of 95 °C for 30 s, 70 °C for 20 s, and 63 °C for 60 s; and (ii) 35 cycles of 95 °C for 10 s, 53 °C for 20 s, and 73 °C for 20 s. A melting curve step was performed (from 35 °C to 75 °C with temperature increments of 0.5 °C for 3 s) to acquire fluorescence values on all four channels (FAM, ROX, CY5 and HEX). The melting peaks were derived from the melting curve data. In summary, two specific designed PNA oligomers were used in qPCR: one was a clamping PNA, which suppresses the amplification of an undesired or wild-type allele, and the other is a PNA detection probe, which has a fluorophore and a quencher group at each terminus of the Probe. The mutations detected by the melting temperature of each tube of fluorescent dye are summarized in Additional file 1: Table S1. The plasma test was performed by Choi J-J, who was blind to the results obtained from the tissue samples.

### Statistical analysis

The PFS and OS were evaluated using the Kaplan-Meier method. The log-rank test was used to compare the PFS with the OS according to the *EGFR* or *KRAS* mutation status. The 95 % confidence intervals for concordance, sensitivity and specificity were calculated by the Wilson Score method. All tests were two-sided, and *P*-values less than 0.05 were considered statistically significant. All statistical analyses were conducted using SPSS 21 software (IBM SPSS Chicago, IL, USA).

## Results

### Determination of mutation status in the plasma samples

The mutation status was determined with PCR clamping, which can specifically block the chain elongation step on a perfectly matched template (wild type [WT]) without interfering with the templates containing mismatched bases (mutant EGFR or KRAS). PNA detection probes with fluorescent dyes and quencher labels were also used in this technique. This hybridization probe system consists of a pair of probes (Fig. 1a, b). To determine the PANAMutyper™ R EGFR and KRAS sensitivity for each tested mutation, genomic DNA (gDNA) from the mutant cell line containing the tested mutation was serially diluted 5 times into highly concentrated WT gDNA from the human lung cancer cell line A549. The 0.005 and 0.001 % sensitivity values of EGFR and KRAS, respectively, corresponded to a specific detection of 2 and 1 mutated copies in 40,000 copies of WT DNA, respectively (Fig. 1c, d). All experimental points were obtained in triplicate, and all plasma samples that met the inclusion criteria were analyzed correctly (success rate of 100 %).

### Patient characteristics

Of the 289 patients who participated in the randomized phase II study, 208 cases with a known *EGFR* or *KRAS* mutation status in their tumor tissues were examined. The median age was 58 years (range: 29 to 82). Most of the patients were male (65.4 %) and had a history of smoking (63 %) and presented an adenocarcinoma histology (78.8 %), stage IV disease (72.6 %), and a good performance status (PS) of 0 or 1 (72.6 %). All patients received GP or IP as the first-line therapy. Among the 208 patients, 37 had *EGFR* exon 19 deletions (19DEL) or L858R mutations and 12 patients had KRAS codon 12 mutations. A total of 98 patients received EGFR-TKI as a second- or third-line therapy upon disease progression. The characteristics of the study population are shown in Table 1.

**Table 1:** Patient demographics and disease characteristics

Variable	All patients (*n* = 208)	
	No.	%
Age, years		
Median	58	
Range	29–82	
Sex		
Male	136	65.4
Female	72	34.6
Smoking status		
Current	73	35.1
Former	58	27.9
Never	77	37.0
Histology		
Adenocarcinoma	164	78.8
Squamous cell	32	15.4
Other	12	5.8
Stage		
IIIB	15	7.2
IV	193	92.8
ECOG PS		
0	17	8.2
1	134	64.4
2	57	27.4
First-line therapy		
GP	105	50.5
IP	103	49.5
Salvage EGFR-TKI therapy		
Second-line	60	28.8
Third-line	38	18.3
No	110	52.9
EGFR mutations		
19DEL	38	18.3
L858R	13	6.3
Wild type	143	68.7
Not determined	14	6.7
KRAS mutations		
Codon 12	8	3.8
Codon 13	2	1.0
Codon 61	2	1.0
Wild type	123	59.1
Not determined	73	35.1

EGFR-TKI: epidermal growth factor receptor-tyrosine kinase inhibitor

### Tumor tissue and plasma *EGFR* and *KRAS* mutation analyses

Matched tumor tissue and plasma mutation statuses were available for 194 patients for *EGFR* and 135 patients for *KRAS*. A comparison of the *EGFR* and *KRAS* mutation status between the tumor biopsies and plasma samples is summarized in Table 2. The concordance of *EGFR* mutation status between the tumor tissue and plasma was 82 % (95 % confidence interval [CI], 76.5–87.4), and it presented a sensitivity of 66.7 % (95 % CI, 60.0–73.3) and a specificity of 87.4 % (95 % CI, 82.7–92.1). The positive and negative predictive values were 65.4 % (95 % CI, 58.7–72.1) and 88.0 % (83.5–92.6), respectively.

**Table 2:** Comparison of the *EGFR* and *KRAS* mutation status in the tumor tissue and plasma

Plasma *EGFR* mutation status									
Tissue *EGFR* mutation status	Positive				Negative				Total
Positive	34				17				51
Negative	18				125				143
Total	52				142				194
	G719A	19DEL	L858R	T790M	Exon20ins	S768I	Multiple	Wild	Total
G719A									0
19DEL		21	1	1			1	14	38
L858R			9			1		3	13
T790M									0
exon20ins									0
S768I									0
Multiple									0
Wild type		3	3	7	2		3	125	143
Total	0	24	13	8	2	1	4	142	194
	N				% (95 % CI)				
Concordance	159				82.0 (76.5–87.4)				
Sensitivity	34				66.7 (60.0–73.3)				
Specificity	125				87.4 (82.7–92.1)				
PPV	52				65.4 (58.7–72.1)				
NPV	142				88.0 (83.5–92.6)				
Plasma *KRAS* mutation status									
Tissue *KRAS* mutation status	Positive				Negative			Total	
Positive	6				6			12	
Negative	13				110			123	
Total	19				116			135	
	Codon 12	Codon 13	Codon 59		Codon 61	Codon 12/61	Wild	Total	
Codon 12	3						5	8	
Codon 13		1					1	2	
Codon 59									
Codon 61			1		1			2	
Codon 12/61									
Wild type	10				2	1	110	123	
Total	13	1	1		3	1	116	135	
	N				% (95 % CI)				
Concordance	116				85.9 (80.1–91.8)				
Sensitivity	6				50.0 (41.6–58.4)				
Specificity	110				89.4 (84.2–94.6)				
PPV	19				31.6 (23.7–39.4)				
NPV	116				94.8 (91.1–98.6)				

The concordance of *KRAS* mutation status between the tumor tissue and plasma was 85.9 % (95 % CI, 80.1–91.8), and it presented a sensitivity of 50.0 % (95 % CI, 41.6–58.4) and a specificity of 89.4 % (95 % CI, 84.2–94.6). The positive and negative predictive values were 31.6 % (95 % CI, 58.7–72.1) and 94.8 % (83.5–92.6), respectively.

To validate the additional mutations that were identified in only the plasma samples, we tested the plasma samples using a digital PCR assay. Six of 7 T790M and 2 of 3 L858R *EGFR* mutations that were detected in only the plasma samples were confirmed by the digital PCR assay. We were unable to confirm the results in the remaining two samples because the total droplet number in the digital PCR was too low.

### *EGFR* and *KRAS* mutation status and clinical outcome

**Fig. 2:**
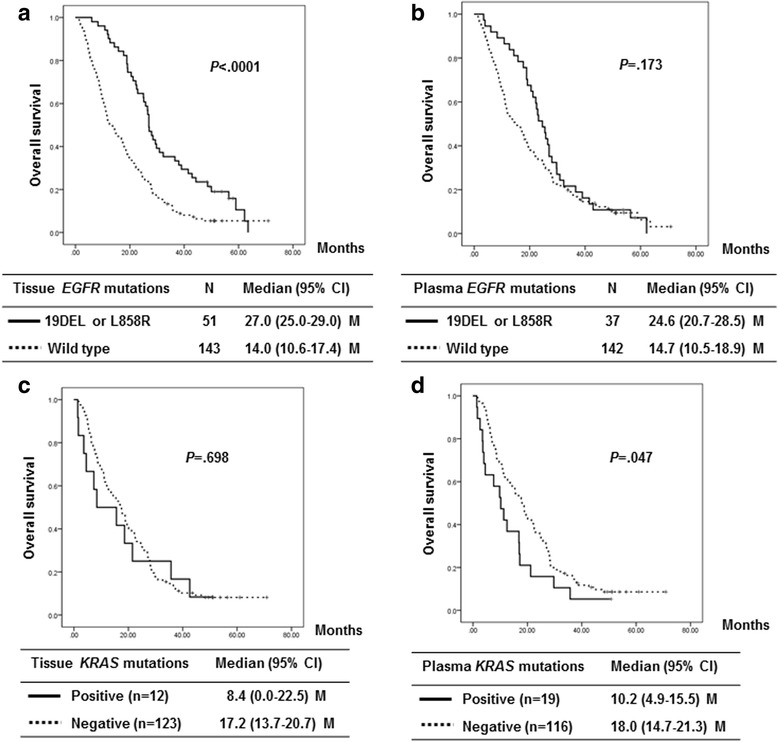
Overall survival according to the *EGFR* mutation status in the tumor tissue (**a**) and plasma (**b**) and the KRAS mutation status in the tumor tissue (**c**) and plasma (**d**)

The cut-off for the OS update was June 29, 2015, and the median duration of follow-up investigations was 16.7 months (range: 0.5 to 70.9 months). The median OS was 24.6 months (95 % CI, 20.7–28.5) in patients with plasma *EGFR* 19DEL or L859R mutations and 27.0 months (95 % CI, 25.0–29.0)) in patients with these mutations in their tumor tissue. The median OS was 10.2 months (95 % CI, 4.9–15.5) in patients with plasma *KRAS* mutations and 8.4 months (95 % CI, 0.0–22.5) in patients with these mutations in their tumor tissue. The Kaplan-Meier curves for the OS according to the tissue and plasma mutation status are shown in Fig. 2.

**Fig. 3:**
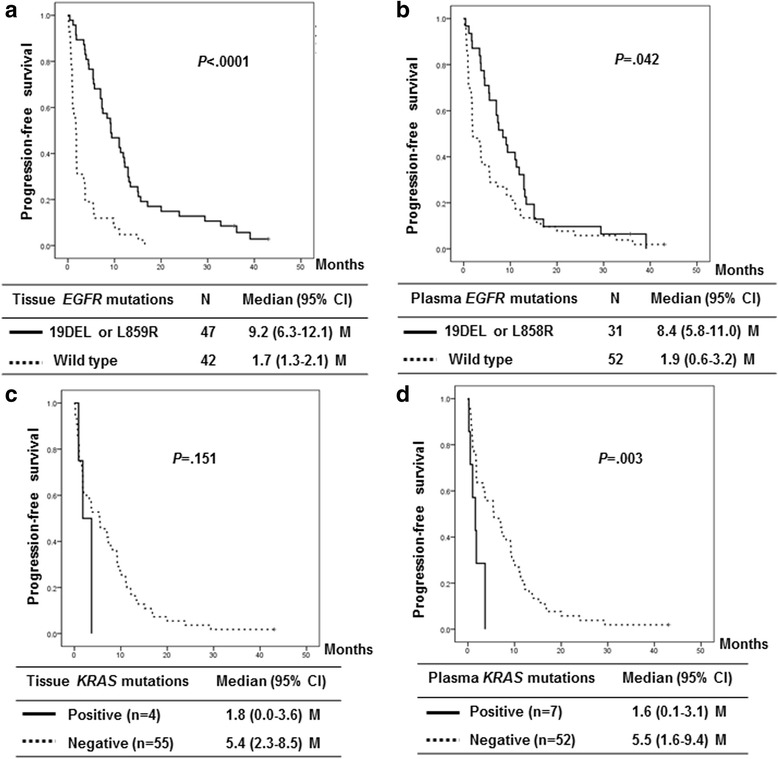
Progression-free survival following EGFR-TKI therapy according to the *EGFR* mutation status in tissue (**a**) and plasma (**b**) and the *KRAS* mutation status in tissue (**c**) and plasma (**d**)

Because *EGFR* and *KRAS* mutations are predictive for the efficacy of EGFR-TKI therapy, we analyzed the association between the mutation status and the therapeutic benefits of this treatment. Of the 208 patients enrolled in this study, 98 received EGFR-TKI therapy as a second- or third-line therapy. We compared the efficacy of EGFR-TKI therapy in the patients carrying common sensitive *EGFR* mutations, including 19DEL and L858R with the patients carrying wild type *EGFR*. The median PFS for EGFR-TKI therapy was 8.4 (95 % CI, 5.8–11.0) months for the patients with plasma *EGFR* 19DEL or L858R mutations and 9.2 (95 % CI, 6.3–12.1) months for the patients with tissue *EGFR* 19DEL or L858R mutations. The median PFS of the patients with plasma *KRAS* mutations was 1.6 months (95 % CI, 0.1–3.1), a result that was comparable with the PFS of patients with tumor-tissue *KRAS* mutations (median PFS, 1.8 months [95 % CI, 0.0–3.6]). The Kaplan-Meier curves for the PFS according to the tissue and plasma mutation status are shown in Fig. 3. However, the correlation of mutations with outcome is of limited interest due to the concordance between mutational status in tissue and plasma.

Patients with additional *EGFR* mutations found in plasma, such as exon20ins and S768I, and multiple mutations (19DEL, L858R, and exon 20ins) showed primary resistance to EGFR-TKI therapy and presented a median PFS of 1.7 months (95 % CI, 0.6–2.8). We also found 8 de novo T790M mutations in plasma samples and three received EGFR-TKI therapy. One patient showed primary resistance and two showed partial responses with PFS values of 9.8 and 15 months.

## Discussion

This report describes the first demonstration of a PNA clamping-assisted fluorescence melting curve analysis for the detection of *EGFR* and *KRAS* mutations in the plasma cfDNA of NSCLC patients. Our data revealed a relatively high concordance (82.0 % for *EGFR* and 85.9 % for *KRAS*) and specificity (87.4 % for *EGFR* and 89.4 % for *KRAS*) compared with other reported techniques such as the amplification refractory mutation systems, denaturing high-performance liquid chromatography, and PNA-PCR [23]. The *EGFR* mutation status has clinical significance as a biomarker and can be used to determine the best treatment for advanced NSCLC; thus, most studies that have evaluated novel analysis techniques have focused on detecting EGFR mutations in blood samples from NSCLC patients. The best reported data for patients with NSCLC were obtained with an EGFR mutation test that uses digital PCR, which resulted in a 92 % sensitivity and 100 % specificity [10]; however, the authors used only 35 samples from NSCLC patients to evaluate the accuracy of the technique. Recently, Douillard et al. reported a high global concordance (94.3 %), specificity (99.8 %) and sensitivity (67.5 %) of the Scorpion ARMS-based EGFR detection kit using 652 samples from patients with advanced NSCLC [24], and the sensitivity was similar to our results (66.7 %). Because of its low sensitivity, the plasma EGFR mutation status appeared to be less predictive for EGFR-TKI therapy benefits than the tissue EGFR mutation status (Fig. 3a, b). The sensitivity of test can be dependent on the condition of samples. The low sensitivity in our study may be attributed from long period of sample storage and multiple freeze and thaw cycles. The lower concordance observed in our study resulted from the additional detection of rare *EGFR* mutations in the plasma samples that were not detected using routine methods. We were able to validate 8 of 10 additional *EGFR* mutations using the digital PCR assay. Two mutations identified in the plasma samples could not be validated because the sample was insufficient (total droplet number was too low for the digital PCR). Unfortunately, additional matched tissue samples were unavailable for testing. However, patients with additional *EGFR* mutations, such as exon20ins and S768I, and multiple mutations (19DEL, L858R, and exon 20ins) showed primary resistance to EGFR-TKI therapy with a median PFS of 1.7 months (95 % CI, 0.6–2.8). These findings indicate that the additional rare EGFR mutations found in only the plasma samples may be true positive results. In our study, 8 de novo T790M mutations were found in the plasma samples only. However, the clinical impact of the de novo T790M mutation on EGFR-TKI therapy is complicated. Previously, we analyzed 124 EGFR-mutant NSCLC cases using mass spectrometry and identified 31 (25 %) patients with de novo T790M mutations. Although the patients with the de novo T790M mutations showed a shorter time to progression (TTP) following EGFR-TKI therapy than those without this mutation, significant differences were not observed in the response rate (RR). The median TTP and RR of the patients with the de novo T790M mutations were 6.3 months and 72 %, respectively. Furthermore, the de novo T790M mutations showed a dose-dependent effect on the efficacy of EGFR-TKI therapy [25]. Thus, it is difficult to conclude whether the additional T790M mutations found in only the plasma are true positives. Considering the clinical relevance of T790M mutations with respect to disease progression after EGFR-TKI therapy, further confirmation of the accuracy of T790M mutation detection using our method is required.

To date, most of the blood samples used in KRAS mutation tests have been obtained from colorectal cancer (CRC) patients [14–17]. Thierry et al. reported that a quantitative PCR-based method exhibited 92 % sensitivity and 98 % specificity for CRC [26]. Very recently, Sacher et al. reported that plasma ddPCR exhibited high specificity of 100 % (62 of 62) but modest specificity of 64 % (16 of 25) for the detection of KRAS G12X in lung cancer patients [7]. In our study, the concordance, specificity and sensitivity of the KRAS mutation test were 85.9, 89.4, and 50.0 %.

Mutation analyses of circulating tumor DNA (ctDNA) require highly sensitive techniques because of the small fraction of tumor-specific DNA relative to background levels of normal cfDNA. The sensitivity of conventional analytical methods, such as Sanger sequencing, is not sufficient to detect low-frequency variants. Recent advances in genomics technologies have provided new opportunities for analyzing ctDNA. Therefore, advanced technologies, such as PANAMutyper, BEAMing, castPCR, NGS and digital PCR, can be of clinical utility because they can identify multiple mutations with high sensitivity. These advanced technologies are extremely sensitive (0.01–5 % limit of detection) and suitable for analyzing circulating ctDNA in cancer patients; however, each technology has advantages and disadvantages. The advanced technologies that are currently deployed for analyzing circulating ctDNA in cancer patients are summarized in Table 3. The technologies using digital PCR, such as droplet-based systems and Beads, Emulsions, Amplification and Magnetics (BEAMing), provide quantitative analyses and single-molecule amplification. However, these methods are expensive, have longer assay times and can detect only a limited set of mutations [6, 17, 26–29]. Next-generation sequencing (NGS) technologies have the potential to provide cost-effective alternatives for high-throughput analyses of multiple mutations over wider genomic regions. However, NGS is less sensitive and more complex than other technologies and requires an expensive system and longer assay times [30]. PANAMutyper™ and castPCR are capable of effectively removing background wild-type DNA [18, 22, 26]; therefore, these techniques can detect a single copy of mutant DNA. Moreover, these approaches can be completed within 3 h, and the analysis can be performed with only a real-time PCR instrument; thus, additional specialized or expensive equipment is not required. Although the castPCR system cannot simultaneously genotype multiple mutations [22, 26], the PANAMutyper™ can simultaneously perform multiple mutation detections and genotype determinations, and these outstanding abilities are realized through the use of a PNA clamping-assisted fluorescence melting curve analysis [18].

**Table 3:** Comparison of the advanced technologies deployed for circulating tumor DNA

Product	Mutyper	BEAMing	castPCR	NGS	Digital PCR
Technology	PNA-based mutant enriched PCR and melting curve analysis	Digital PCR and flow cytometry	TaqMan-based mutant enriched PCR	Next generation sequencing	Droplet digital PCR
Sample	10 ng (plasma, 1–2 ml)	(plasma, 2 ml)	10 ng	10–250 ng	10 ng
Genotyping	YES	YES	YES	YES	YES
Multiplex	YES	YES	NO	YES	NO
Running time/Workflow	<3 h/Sample	10 days/complicated	<3 h/sample	2 days/complicated	2 days/complicated
Machine	Real time PCR	Droplet digital PCR, Flow cytometry	Real time PCR	Library machine/PCR/NGS sequencer	Droplet digital PCR/Droplet generator
Sensitivity	0.1–0.01 %	0.1–0.01 %	0.1 %	1–5 %	0.1–0.01 %
Advantages	Only a real-time PCR system is required; higher sensitivity, specificity, and reproducibility; multiplexing; and short run time.	Quantitative analysis; multiplexing	Requires only a real-time PCR system; and short run time.	Multiplexing (target gene panel); Barcoding samples; Quantitative analysis; Detects de novo mutations.	Quantitative analysis
Disadvantages	Cannot detect novel mutations	Cannot detect novel mutations; requires an expensive system; and a longer assay time.	Cannot detect novel mutations or perform multiplexing.	Requires an expensive system; and longer assay time.	Requires an expensive system; longer assay time; and cannot detect de novo mutations.

## Conclusions

We demonstrated that plasma *EGFR* and *KRAS* mutation testing using PNA-based real-time PCR methods is feasible and can be applied as an alternative to tumor genotyping when deciding upon the optimal treatment of NSCLC patients. Further studies are required to increase the testing accuracy and determine the clinical applications of this technique.

## Additional file

Additional file 1: Table S1. The criteria of the mutation detection according to the fluorescent dye and melting temperature. (DOCX 25 kb)

## Abbreviations

BEAMing: Beads, Emulsions, Amplification and Magnetics
cfDNA: Circulating cell-free DNA
ctDNA: Circulating Tumor DNA
EGFR: Epidermal Growth Factor Receptor
FFPE: Formalin-fixed, Paraffin-embedded
GP: Gemcitabine and Cisplatin
IP: Irinotecan and Cisplatin
NSCLC: Non-small Cell Lung Cancer
OS: Overall Survival
PCR: Polymerase Chain Reaction
PFS: Progression-free Survival
PNA: Peptide Nucleic Acid
TKI: Tyrosine Kinase Inhibitor
