# Erratum to: PNA clamping-assisted fluorescence melting curve analysis for detecting *EGFR* and *KRAS* mutations in the circulating tumor DNA of patients with advanced non-small cell lung cancer

**DOI:** 10.1186/s12885-016-2760-9

**Published:** 2016-10-17

**Authors:** Ji-Youn Han, Jae-Jin Choi, Jin Young Kim, You Lim Han, Geon Kook Lee

**Affiliations:** 1 Lung Cancer Branch, Research Institute, National Cancer Center, Goyang, Korea; 2 PANAGENE Inc., Daejeon, Korea; 3 Center for Lung Cancer, Hospital, National Cancer Center, 323 Ilsan-ro, Ilsan-dong-gu, Goyang, Gyeonggi 10408 Korea

## Erratum

Unfortunately, the original version of this article [1] contained an error. Within Fig. 1, Fig. 1c and d were missing. The correct version of Fig. 1 can be found below and has been updated in the original article.

**Fig. 1:**
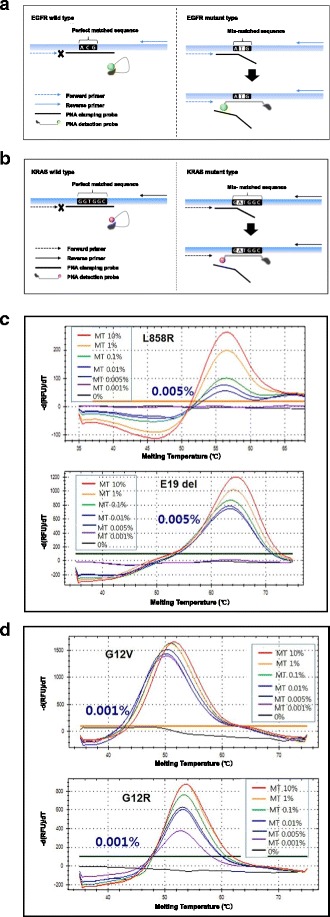
Schematic representation of EGFR and KRAS mutation detection using PANAMutyperTM: EGFR (**a**) and KRAS (**b**). Sensitivity of the EGFR L858R and E19del (**c**) and KRAS G12V and G12R (**d**) mutants according to their cellularity by diluting to 100, 10, 1, 0.1, 0.01, 0 % with respect to the wild cell line DNA and mutant cell line DNA. The data presented here are representative obtained from sensitivity test conducted more than 50 times. MT, mutant type
