# Supplementary material for: PNA clamping-assisted fluorescence melting curve analysis for detecting EGFR and KRAS mutations in the circulating tumor DNA of patients with advanced non-small cell lung cancer
Source: BMC Cancer. 2016 Aug 12;16:627. doi: 10.1186/s12885-016-2678-2 (PMC4983013; doi:10.1186/s12885-016-2678-2)
Supplement: Additional file 1: Table S1. — The criteria of the mutation detection according to the fluorescent dye and melting temperature. (DOCX 25 kb) [file 12885_2016_2678_MOESM1_ESM.docx]

Table S1. The criteria of the mutation detection according to the fluorescent dye and melting temperature

<EGFR>

| Reagent | Fluorescent Dye | Melting Temperature | Assessment | |
| --- | --- | --- | --- | --- |
|  |  |  | Amino Acid Change | Nucleotide change |
| G719X | FAM | 57℃ ~ 60℃ | p.G719A | c.2156G>C |
|  |  | 46℃ ~ 48.5℃ | p.G719S | c.2155G>A |
|  |  | 50℃ ~ 53℃ | p.G719C | c.2155G>T |
| E19delA & B | HEX | 58℃ ~ 67℃ | p.E746_A750del | c.2235_2249 del 15 |
|  |  |  | p.E746_A750del | c.2236_2250 del 15 |
|  |  |  | p.E746_T751>IP | c.2235_2251 >AATTC |
|  |  |  | p.K745_E749del | c.2233_2247 del15 |
|  |  |  | p.E746_T751>I | c.2235_2252 AAT (complex) |
|  |  |  | p.E746_A750del | c.2235_2248 >AATTC |
|  |  |  | p.L747_S752>Q | c.2239_2256 >CAA |
|  |  |  | p.S752_I759del | c.2253_2276 del 24 |
|  |  |  | p.E746_T751>VA | c.2237_2253>TTGCT |
|  |  |  | p.E746_T751>A | c.2237_2251 del 15 |
|  |  |  | p.L747_T751del | c.2239_2253 del 15 |
|  |  |  | p. L747_T751del | c.2238_2252 del 15 |
|  |  |  | p.L747_T751>Q | c.2238_2252 >GCA(complex) |
|  |  |  | p.L747_T751del | c.2240_2254 del 15 |
|  |  |  | p.E746_T751>V | c.2237_2252 >T |
|  |  |  | p.E746_S752>I | c.2235_2255 >AAT |
|  |  |  | p.E746_T751del | c.2236_2253 del 18 |
|  |  |  | p.E746_S752>A | c.2237_2254 del 18 |
|  |  |  | p.E746_S752>V | c.2237_2255>T (complex) |
|  |  |  | p.E746_S752>D | c.2238_2255 del 18 |
|  |  |  | p.L747_A750>P | c.2238_2248 >GC(complex) |
|  |  |  | p.L747_E749del | c.2239_2247 del 9 |
|  |  |  | p.L747_S752del | c.2239_2256 del 18 |
|  |  |  | p.L747_ A750>P | c.2239_2248 TTAAGAGAAG>C |
|  |  |  | p.L747_P753>Q | c.2239_2258 >CA(complex) |
|  |  |  | p.L747_T751>S | c.2240_2251 del 12 |
|  |  |  | p.L747_P753>S | c.2240_2257 del 18 |
|  |  |  | p.L747_T751>P | c.2239_2251 >C(complex) |
|  |  |  | p.E746_P753>VS | c.2237_2257 >TCT |
| S768I | HEX | 58℃ ~ 62℃ | p.S768I | c.2303G>T |
| T790M | HEX | 58℃ ~ 62℃ | p.T790M | c.2369C>T |
| E20insA&B | ROX | 60℃ ~ 69℃ | p.D770_N771insG | c.2310_2311 insGGT |
|  |  |  | p.P772_H773insTTP | c.2315_2316 insGACAACCCC |
|  |  |  | p.P772_H773insGNP | c.2315_2316 insGGGCAACCC |
|  |  |  | p.V769_N770insASV | c.2309_2310 AC>CCAGCGTGGAT |
|  |  |  | p.V769_D770insASV | c.2307_2308 ins9 GCCAGCGTG |
|  |  |  | p.H773_V774insH | c.2319_2320 insCAC |
|  |  |  | p.H773L | c.2318 A>T |
|  |  |  | p.H773_V774insPH | c.2319_2320 insCCCCAC |
|  |  |  | p.V774_C775insHV | c.2321_2322 insCCACGT |
|  |  |  | p.D770_N771insSVD | c.2311_2312 ins9 GCGTGGACA |
| L858R | ROX | 55.5℃ ~ 59.5℃ | L858R | c.2573T>G |
|  |  | 44℃ ~ 47℃ | L858R | c.2573_2574 TG>GT |
| L861Q | ROX | 48.5℃ ~ 53.5℃ | L861Q | c.2582T>A |
| EIC | Cy5 | 59℃~63℃ | Internal control | |

<KRAS>

| Reagent | Fluorescent Dye | Melting Temperature | Assessment | |
| --- | --- | --- | --- | --- |
|  |  |  | Amino Acid Change | Nucleotide change |
| KC12a | HEX | 46.5℃ ~ 50.5℃ | p.G12A | c.35G>C |
|  | ROX | 38.5℃ ~ 42.5℃ | p.G12V | c.35G>T |
|  | HEX | 51℃ ~ 55℃ | p.G12R | c.34G>C |
|  | ROX | 43℃ ~47℃ | p.G12C | c.34G>T |
| KC12b | FAM | 46.5℃ ~50.5℃ | p.G12D | c.35G>A |
|  | FAM | 51.5℃ ~55.5℃ | p.G12S | c.34G>A |
| KC13a | FAM | 47.5℃ ~51.5℃ | p.G13A | c.38G>C |
|  | Cy5 | 51.5℃ ~55.5℃ | p.G13V | c.38G>T |
|  | FAM | 43℃ ~47℃ | p.G13R | c.37G>C |
|  | FAM | 38.5℃ ~ 42.5℃ | p.G13C | c.37G>T |
| KC13b | FAM | 44.5℃ ~ 48.5℃ | p.G13D | c.38G>A |
|  | ROX | 47℃ ~ 53℃ | p.G13S | c.37G>A |
| KC59 | ROX | 50℃ ~ 54℃ | p.A59T | c.175G>A |
|  | HEX | 43.5℃ ~47.5℃ | p.A59E | c.176C>A |
|  | HEX | 53℃ ~ 57℃ | p.A59G | c.176C>G |
| KC61 | HEX | 41.5℃ ~ 45.5℃ | p.Q61K | c.181C>A |
|  | HEX | 51.5℃ ~ 55.5℃ | p.Q61E | c.181C>G |
|  | FAM | 42.5℃ ~ 46.5℃ | p.Q61P | c.182A>C |
|  | HEX | 46℃ ~ 50℃ | p.Q61R | c.182A>G |
|  | FAM | 52℃ ~ 56℃ | p.Q61L | c.182A>T |
|  | ROX | 42.5℃ ~ 48.5℃ | p.Q61H | c.183A>C |
|  | ROX | 49℃ ~ 55℃ | p.Q61H | c.183A>T |
| KC117 | FAM | 42℃ ~ 46℃ | [p.K117E](http://cancer.sanger.ac.uk/cosmic/mutation/overview?id=1360831) | [c.349A>G](http://cancer.sanger.ac.uk/cosmic/mutation/overview?id=1360831) |
|  | HEX | 41.5℃ ~ 45.5℃ | [p.K117N](http://cancer.sanger.ac.uk/cosmic/mutation/overview?id=19940) | [c.351A>C](http://cancer.sanger.ac.uk/cosmic/mutation/overview?id=19940) |
|  | ROX | 40.5℃ ~ 44.5℃ | [p.K117N](http://cancer.sanger.ac.uk/cosmic/mutation/overview?id=28519) | [c.351A>T](http://cancer.sanger.ac.uk/cosmic/mutation/overview?id=28519) |
| KC146 | HEX | 44.5℃ ~ 48.5℃ | [p.A146T](http://cancer.sanger.ac.uk/cosmic/mutation/overview?id=19404) | [c.436G>A](http://cancer.sanger.ac.uk/cosmic/mutation/overview?id=19404) |
|  | HEX | 51.5℃ ~ 55.5℃ | [p.A146P](http://cancer.sanger.ac.uk/cosmic/mutation/overview?id=19905) | [c.436G>C](http://cancer.sanger.ac.uk/cosmic/mutation/overview?id=19905) |
|  | FAM | 52℃ ~ 56℃ | [p.A146G](http://cancer.sanger.ac.uk/cosmic/mutation/overview?id=1360829) | [c.437C>G](http://cancer.sanger.ac.uk/cosmic/mutation/overview?id=1360829) |
|  | FAM | 43℃ ~ 47℃ | [p.A146V](http://cancer.sanger.ac.uk/cosmic/mutation/overview?id=19900) | [c.437C>T](http://cancer.sanger.ac.uk/cosmic/mutation/overview?id=19900) |
| KIC | HEX | 54℃ ~ 58℃ | Internal control | |
